# Persistence of Telemedicine Usage for Breast and Prostate Cancer after the Peak of the COVID-19 Pandemic

**DOI:** 10.3390/cancers15204961

**Published:** 2023-10-12

**Authors:** Susan Chimonas, Allison Lipitz-Snyderman, Zoe Spiegelhoff, Nirjhar Chakraborty, Kenneth Seier, Charlie White, Gilad Kuperman

**Affiliations:** 1 Department of Epidemiology and Biostatistics, Memorial Sloan Kettering Cancer Center, New York, NY 10065, USAchakran@mskcc.org (N.C.); seierk@mskcc.org (K.S.); whitec4@mskcc.org (C.W.); 2 Department of Health Informatics, Memorial Sloan Kettering Cancer Center, New York, NY 10065, USA; 3 Department of Informatics, Memorial Sloan Kettering Cancer Center, New York, NY 10065, USA; kupermag@mskcc.org

**Keywords:** telemedicine, oncology, breast cancer, prostate cancer, remote care

## Abstract

**Simple Summary:**

Telemedicine became widespread during the COVID-19 pandemic, but little is known about its persistence in routine cancer care after the pandemic’s peak. This study examined telemedicine use for breast and prostate cancer patients at a New York City cancer center before, during, and after the pandemic’s peak. Telemedicine usage increased from 2% before the pandemic to 50% during the peak and then decreased to 30% after the peak. Both during and after the peak, psychiatry, social work, and nutrition conducted almost all visits remotely, while surgery and nursing maintained low telemedicine usage. Most departments continued to use telemedicine at or above peak levels, except for medicine, neurology, and survivorship. Anesthesiology and neurology used telemedicine more for follow-ups, while nursing used it more for new visits. These findings highlight specific contexts where patients and providers choose telemedicine even when other options are available. However, more research is needed to assess telemedicine’s suitability for and impact on cancer care.

**Abstract:**

While COVID-19 catalyzed a shift to telemedicine, little is known about the persistence of remote cancer care in non-emergent times. We assessed telemedicine use at a high-volume academic cancer center in New York City and analyzed breast and prostate cancer visits pre-COVID-19, peak COVID-19, and post-peak. Descriptive statistics assessed visit mode (in person, telemedicine) and type (new, follow-up, other) by department/specialty, with Fisher’s exact tests comparing peak/post-peak differences. The study included 602,233 visits, with telemedicine comprising 2% of visits pre-COVID-19, 50% peak COVID-19, and 30% post-peak. Notable variations emerged by department/specialty and visit type. Post-peak, most departments/specialties continued using telemedicine near or above peak levels, except medicine, neurology, and survivorship, where remote care fell. In psychiatry, social work, and nutrition, nearly all visits were conducted remotely during and after peak COVID-19, while surgery and nursing maintained low telemedicine usage. Post-peak, anesthesiology and neurology used telemedicine seldom for new visits but often for follow-ups, while nursing showed the opposite pattern. These trends suggest department- and visit-specific contexts where providers and patients choose telemedicine in non-emergent conditions. More research is needed to explore these findings and evaluate telemedicine’s appropriateness and impact across the care continuum.

## 1. Introduction

Telemedicine holds great promise for augmenting and enhancing the delivery of cancer care across the continuum, improving patient access, convenience, and overall quality of care. However, the contexts in which cancer patients and providers select remote care remain relatively unexplored. Only a few studies have examined these issues, mostly focused on a single specialty, patient population, or treatment scenario (e.g., neurosurgery, radiotherapy) [1,2,3,4,5,6,7,8]. What is clear, however, is that telemedicine “is not ideally suited for all patients and/or clinical scenarios, for a wide range of reasons” [9]. Better understanding patterns in telemedicine’s use is therefore crucial for optimizing its integration and maximizing its benefits.

The COVID-19 pandemic provides unique opportunities to explore these vital issues. While few oncologists in the U.S. used telemedicine pre-pandemic, the emergence of COVID-19 obliged them to deliver as much care as possible via telemedicine—by necessity, even when in-person visits might have been more appropriate or optimal [9,10,11,12]. As in-person options have returned, post-peak care patterns can indicate situations where telemedicine has persisted as a part of routine care.

Leveraging these data, this study assessed trends in telemedicine use for breast and prostate cancer patients at a high-volume academic cancer center in New York City before, during, and after the peak of the COVID-19 pandemic. We hypothesized that trends by department/specialty and visit type—particularly in the post-peak period—could distinguish specific contexts in which telemedicine was frequently chosen by providers and patients after in-person options re-emerged. The findings offer new insights around the use of telemedicine in routine cancer care and suggest promising areas for future research into telemedicine appropriateness.

## 2. Materials and Methods

Setting: This retrospective study analyzed trends in telemedicine utilization at Memorial Sloan Kettering Cancer Center (MSK), a high-volume academic cancer center in New York City. The study period was divided into three phases: pre-COVID-19 (March 2019 to February 2020), peak COVID-19 (March 2020 to February 2021), and post-peak (March 2021 to February 2022). We focused on visits by breast and prostate cancer patients, the two most common non-dermatologic malignancies in women and men [13,14,15]. Our sample was part of a larger study of these populations, as reviewed and approved by MSK’s Institutional Review Board.

Data sources: Data were obtained from electronic health records (EHR) and administrative databases for clinical visits deemed to be feasible by telemedicine, meaning they did not require diagnostic studies, treatments, or procedures. Data captured for each visit included department/specialty, visit mode (telephone, video, or in person), and visit type (new, follow-up, or other). As recorded in the EHR, “new visits” included appointments for patients visiting MSK for the first time, as well as for established patients seeing a new department/specialty. Thus, the “new visits” category spanned the continuum of care, from patients’ initial appointment at MSK to developments anywhere along the continuum where additional departments/specialties became involved. “Follow-up visits” were those in which established patients were returning to a department/specialty for additional consultations. “Other” visits encompassed a heterogeneous range of services labeled as neither “new” nor “follow-up” in the EHR. We also captured the total number of unique patients in the 3 study periods, as well as their demographic characteristics (age, race, ethnicity, gender, cancer type, primary language).

Data analysis: We combined telephone and video visits into a single category of “telemedicine visits.” We used descriptive statistics to assess frequencies of telemedicine versus in person, as well as new, follow-up, and other visits by specialty/department over time. We used Fishers’ exact tests to assess significant changes in usage patterns from the peak to post-peak period. A *p*-value < 0.05 was considered statistically significant for these analyses. Analyses were conducted using the R software program (version 4.3.1).

## 3. Results

The study included a total of 602,233 visits, of which 26% (158,986) were conducted via telemedicine. We found no notable differences in the numbers of patients or visits over time, and patients’ characteristics were also comparable across the pre-COVID-19, peak-COVID-19, and post-peak periods (Table 1).

Institution-level trends: As to be expected, we found significant fluctuations in the usage of telemedicine across the three study periods overall: During the pre-COVID-19 period, telemedicine accounted for only 2% (3344) of feasible visits. However, with the onset of the pandemic, there was a dramatic shift towards telemedicine, with utilization reaching 50% (89,915) during the peak COVID-19 time-frame. Subsequently, in the post-peak period, telemedicine utilization decreased to 30% (65,727) as in-person care regained prominence (Table 2).

**Figure 1:**
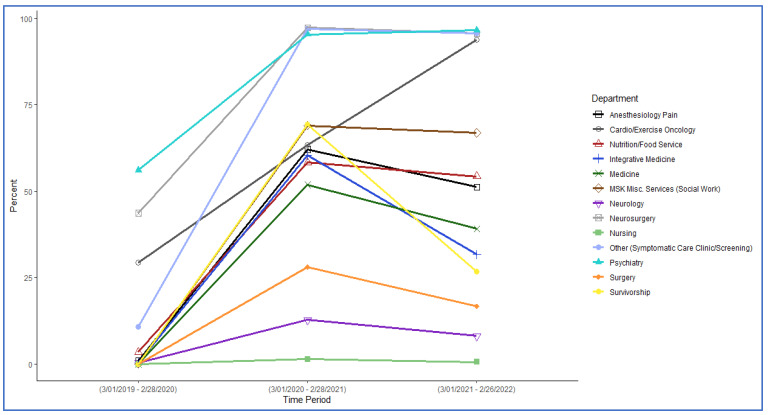
Telemedicine usage by department before, during, and after the pandemic’s peak.

Trends by department/specialty: We also found striking temporal variations by department/specialty. In departments such as anesthesiology, integrative medicine, and neurosurgery, the majority of all feasible visits were conducted via telemedicine during peak COVID-19, and this pattern persisted post-peak. Indeed, in cardiology, nutrition, psychiatry, and social work, nearly all visits (94%, 96%, 96%, and 97%, respectively) continued to be conducted remotely post-peak. Conversely, nursing and surgery demonstrated the lowest rates of growth in telemedicine utilization, with post-peak telemedicine rates of only 8% and 17%, respectively. Medicine, neurology, and survivorship showed relatively robust telemedicine growth during peak COVID-19, but usage dropped significantly post-peak to 32%, 39%, and 27%, respectively (Table 2, Figure 1). 

Trends by visit type: While few visits of any kind were conducted remotely pre-COVID-19, we observed variations in peak and post-peak telemedicine utilization by visit type. During the peak period, about half of new and follow-up visits, and nearly all other visits, occurred via telemedicine. Post-peak, less than a third of new and follow-up visits (8647 and 49,527, respectively) occurred remotely, but nearly all other visits continued to be conducted via telemedicine (Table 3). These visit-type usage patterns further diverged by department/specialty (Table 3). For instance, post-peak, few visits of any type occurred via telemedicine in surgery and survivorship. However, cardiology, nutrition, and psychiatry conducted the vast majority (in excess of 70%) of new, follow-up, and other visits by telemedicine. Integrative medicine, medicine, and social work also showed frequent telemedicine use (30% or greater) for all visit types. In anesthesiology and neurology, telemedicine was rare for new and other visits but common for follow-ups. In contrast, nursing showed the opposite pattern, with telemedicine used very often for new and other visits but rarely for follow-up consultations.

## 4. Discussion

Appropriately leveraged, telemedicine can enhance patient access, improve care coordination, and provide patient-centered services, but few studies have identified the acceptable contexts for remote cancer care. To this end, our study investigated trends in telemedicine utilization for breast and prostate cancer patients at a high-volume academic cancer center before, during, and after the peak of COVID-19.

Overall, we found an exponential, institution-wide increase in telemedicine visits during the peak COVID-19 period to the point where about half of feasible encounters were being delivered remotely. This indicates the adaptability of telemedicine in delivering care during emergencies and its potential to ensure the continuity of care in challenging circumstances. In the post-peak period, the proportion of feasible visits conducted via telemedicine dropped significantly but was still 30%, well above the pre-pandemic baseline. This is consistent with national trends, in which the arrival of vaccines, relaxation of restrictions, improved safety measures, policy changes, and patient and clinician preferences influenced this shift back to traditional care delivery methods [1,16,17,18,19,20]. In addition, as we hypothesized, the pandemic likely highlighted for many clinicians and patients numerous clinical contexts where remote care was suitable.

The specific decision about when to have an encounter via telemedicine as opposed to in person involves a multi-factorial set of considerations, including the goals of the encounter, time and cost to patients of an in-person experience, patient comfort with technology, and patient and physician preferences. Our data show that patients and providers are continuing to choose remote interactions as an acceptable and perhaps even preferable option. Understanding how each of these factors plays into the decision should be the focus of future research.

We also found dramatic variations by specialty and visit type in the proportion of visits provided remotely peak and post-peak. For instance, we found that the post-peak decline in telemedicine was mostly driven by drops in utilization by the departments of medicine, neurology, and survivorship; most other departments/specialties continued telemedicine use near or above peak frequencies. Psychiatry, social work, and nutrition departments, especially, demonstrated very high proportions of telemedicine visits both during and after the peak COVID-19 period. This finding suggests that these services successfully and widely embraced telemedicine as a means to deliver routine counseling, support, and education remotely—results that align with other studies [1,19,20,21,22]. In contrast, departments such as surgery and nursing showed relatively limited growth in telemedicine utilization during the peak period and correspondingly modest declines post-peak. This is likely due to the procedural and hands-on nature of care in these departments, which largely necessitate in-person visits for examinations, interventions, and complex treatments. These diverse findings underscore the importance of tailoring telemedicine strategies to match the specific needs and challenges faced by different specialists and their patients, as highlighted by prior studies [22,23]. Further research should examine this variability in greater depth to determine if there are best practices for telemedicine use that could be disseminated across institutions.

Our findings also suggest that telemedicine is used variably across the cancer care continuum. In other studies, it has been used effectively for follow-up and supportive care [1,19,24,25,26,27,28]. Similarly, we observed high utilization in departments like psychiatry, social work, and nutrition. These services largely involve ongoing communication, counseling, and education, which can often be effectively delivered through remote consultations. On the other hand, departments such as surgery and nursing, which often involve hands-on assessments and interventions at the diagnostic, treatment, and survivorship phases of care, may have more limited potential for telemedicine. Future studies are needed to understand how best to optimize the integration of telemedicine across the cancer care continuum.

Our study also found that departmental telemedicine use patterns varied significantly by visit type. Some departments showed relatively frequent telemedicine use for new, follow-up, and other visits, indicating that remote care is often an acceptable option in many contexts in these specialties. However, several departments diverged from this trend. Surgery and survivorship, for instance, rarely conducted any visit types remotely during or after the pandemic’s peak—suggesting in-person care is almost always considered by providers and patients to be optimal in these specialties. In anesthesiology and neurology, telemedicine visits were common only for follow-ups—reflecting a lesser need for in-person care among established patients receiving ongoing care. Nursing showed the opposite pattern, with telemedicine rarely used for follow-up consultations. Taken together, these diverse patterns extend prior findings [7,29,30,31,32,33,34] by suggesting important department- and visit-specific contexts across the continuum where telemedicine may be suitable.

There are some limitations to consider. First, we use post-peak telemedicine use as an indicator for acceptability; however, true acceptability might differ, and additional factors should be considered when making these determinations. Second, the visits included in the “other” visit category are diverse and include many different types of care. Future work is needed to better understand these visit categories and identify more nuanced clinical contexts for continued telemedicine use. Third, our data are from a single institution, and telemedicine use might be dictated by several factors such as decisions at the leadership level and technology availability.

Understanding the patterns of telemedicine use in different circumstances is crucial to determining how it might best be folded into routine care. To this end, our study identified specific variations in telemedicine use by department and visit type, suggesting distinct contexts where remote care may be frequently selected by patients and providers even when in-person options are available. Further research is warranted to explore the reasons behind these observed variations, identify context-specific barriers to telemedicine adoption, and assess patient and provider satisfaction with remote care delivery. These insights can inform the development of tailored telemedicine strategies that align with specific needs and contexts across the care continuum to enhance oncology care quality, access, cost-effectiveness, and ultimately patient outcomes.

## 5. Conclusions

This study highlights the disparate patterns of telemedicine adoption across different specialties and visit types for breast and prostate cancer patients, reflecting the diverse nature of oncology care. Future research is needed to explore the appropriate contexts for remote care across the continuum and to tailor telemedicine implementation strategies accordingly.

## Figures and Tables

**Table 1:** Patient characteristics, by time period.

Characteristics	Pre-COVID-19 3/2019–2/2020 N = 50,585		Peak COVID-19 3/2020–2/2021 N = 48,522		Post-Peak 3/2021–2/2022 N = 55,612	
Age (yrs)	No.	%	No.	%	No.	%
Age (yrs)						
0–18	12	0%	11	0%	15	0%
19–29	179	0%	171	0%	169	0%
30–39	1565	3%	1496	3%	1691	3%
40–49	6175	12%	5817	12%	6352	11%
50–59	12,572	25%	11,926	25%	13,298	24%
60–69	16,126	32%	15,630	32%	17,849	32%
70–79	12,259	24%	11,992	25%	14,315	26%
80–89	3358	7%	3227	7%	3959	7%
90+	333	1%	307	1%	353	1%
Sex						
Female	34,058	67%	32,747	67%	37,061	67%
Male	16,527	33%	15,775	33%	18,551	33%
Cancer Type						
Breast	34,270	68%	32,908	68%	37,250	67%
Prostate	16,315	32%	15,614	32%	18,362	33%
Ethnicity						
Not Hispanic or Latino	44,522	88%	42,739	88%	48,602	87%
Hispanic or Latino	3164	6%	3143	6%	3707	7%
Missing	2899	6%	2640	5%	3303	6%
Race						
White	38,921	77%	37,195	77%	42,168	76%
Black or African American	4349	9%	4372	9%	5042	9%
Asian or Indian	3395	7%	3289	7%	3986	7%
Other	1434	3%	1420	3%	1725	3%
Missing	2486	5%	3275	7%	2691	5%
Primary Language						
English	47,186	93%	45,784	94%	52,575	95%
Spanish	757	1%	710	1%	809	1%
Russian	477	1%	426	1%	471	1%
Other	2165	4%	2395	5%	1757	3%

**Table 2:** Proportion of telemedicine and in-person consultations over time, by department.

Department/Specialty	Pre-COVID-19 3/2019–2/2020 N = 202,296		Peak COVID-19 3/2020–2/2021 N = 180,649		Post-Peak 3/2021–2/2022 N = 219,288		
	No.	%	No.	%	No.	%	*p*-Value *
Anesthesiology/Pain Mgmt.							
Telemedicine	11	1%	1066	62%	1186	51%	<0.001
In person	1282	99%	653	38%	1132	49%	
Cardio-Oncology/Exercise							
Telemedicine	29	29%	104	63%	527	94%	<0.001
In person	70	71%	60	37%	34	6%	
Integrative Medicine							
Telemedicine	139	3%	1588	58%	2043	54%	0.001
In person	3965	97%	1136	42%	1722	46%	
Medicine							
Telemedicine	383	0%	51,959	60%	31,912	32%	<0.001
In person	89,425	100%	34,205	40%	68,655	68%	
Neurology							
Telemedicine	0	0%	2221	52%	2267	39%	<0.001
In person	4498	100%	2069	48%	3531	61%	
Neurosurgery							
Telemedicine	0	0%	265	69%	347	67%	0.517
In person	409	100%	119	31%	172	33%	
Nursing							
Telemedicine	73	0%	1397	13%	1164	8%	<0.001
In person	17,059	100%	9589	87%	13,190	92%	
Nutrition/Food Service							
Telemedicine	824	44%	2287	97%	2742	96%	0.001
In person	1067	56%	63	3%	126	4%	
Psychiatry							
Telemedicine	566	11%	6965	97%	7665	96%	<0.001
In person	4708	89%	219	3%	352	4%	
Social Work							
Telemedicine	1316	56%	1906	95%	2372	97%	0.045
In person	1033	44%	92	5%	84	3%	
Surgery							
Telemedicine	0	0%	15,028	28%	11,159	17%	<0.001
In person	66,882	100%	38,679	72%	56,130	83%	
Survivorship							
Telemedicine	3	0%	5107	69%	2331	27%	<0.001
In person	7433	100%	2268	31%	6378	73%	
Other							
Telemedicine	0	0%	22	1%	12	1%	0.015
In person	1121	100%	1582	99%	2055	99%	
All							
Telemedicine	3344	2%	89,915	50%	65,727	30%	<0.001
In person	198,952	98%	90,734	50%	153,561	70%	

* Fisher’s exact test comparing peak and post-peak.

**Table 3:** Peak and post-peak telemedicine usage, by visit type and department/specialty.

Department/Specialty	Peak COVID-19 3/2021–2/2022 N = 180,649						Post-Peak 3/2021–2/2022 N = 219,288					
	New		Follow-Up		Other		New		Follow-Up		Other	
	No.	%	No.	%	No.	%	No.	%	No.	%	No.	%
Anesthesiology/Pain Mgmt.												
Telemedicine	46	14%	1020	74%	0	0%	6	1%	1180	63%	0	0%
In person	294	86%	359	26%	0	0%	444	99%	688	37%	0	0%
Cardio/Exercise												
Telemedicine	14	100%	13	100%	77	56%	99	100%	220	100%	208	86%
In person	0	0%	0	0%	60	44%	0	0%	0	0%	34	14%
Integrative Med.												
Telemedicine	552	92%	882	56%	154	28%	834	79%	976	46%	233	41%
In person	49	8%	698	44%	389	72%	225	21%	1163	54%	334	59%
Medicine												
Telemedicine	6077	69%	44,540	59%	1342	100%	4145	38%	26,176	30%	1591	100%
In person	2733	31%	31,466	41%	6	0%	6696	62%	61,959	70%	0	0%
Neurology												
Telemedicine	257	29%	1964	58%	0	0%	133	12%	2134	46%	0	0%
In person	633	71%	1436	42%	0	0%	983	88%	2548	54%	0	0%
Neurosurgery												
Telemedicine	94	75%	171	66%	0	0%	137	71%	208	64%	2	100%
In person	31	25%	88	34%	0	0%	55	29%	117	36%	0	0%
Nursing												
Telemedicine	72	100%	1184	11%	141	60%	73	99%	775	6%	316	80%
In person	0	0%	9495	89%	94	40%	1	1%	13,112	94%	77	20%
Nutrition/Food												
Telemedicine	51	61%	47	60%	2189	100%	377	82%	150	79%	2215	100%
In person	32	39%	31	40%	0	0%	85	18%	41	21%	0	0%
Psychiatry												
Telemedicine	655	95%	5604	97%	706	99%	721	87%	6312	96%	632	99%
In person	38	5%	173	3%	8	1%	111	13%	234	4%	7	1%
Social Work												
Telemedicine	24	43%	26	30%	1856	100%	32	52%	31	36%	2309	100%
In person	32	57%	60	70%	0	0%	30	48%	54	64%	0	0%
Surgery												
Telemedicine	2567	27%	12,442	28%	19	100%	1611	13%	9501	17%	47	100%
In person	6976	73%	31,703	72%	0	0%	10,912	87%	45,218	83%	0	0%
Survivorship												
Telemedicine	911	74%	4196	68%	0	0%	478	30%	1853	26%	0	0%
In person	323	26%	1945	32%	0	0%	1110	70%	5268	74%	0	0%
Other												
Telemedicine	2	29%	20	1%	0	0%	1	9%	11	1%	0	0%
In person	5	71%	1577	99%	0	0%	10	91%	2045	99%	0	0%
All												
Telemedicine	11,322	50%	72,109	48%	6484	92%	8647	30%	49,527	27%	7553	94%
In person	11,146	50%	79,031	52%	557	8%	20,662	70%	132,447	73%	452	6%

## Data Availability

Data are available from the authors upon request.
